# Brazil: the emerging epicenter of COVID-19 pandemic

**DOI:** 10.1590/0037-8682-0550-2020

**Published:** 2020-10-21

**Authors:** Mariane Barros Neiva, Isabelle Carvalho, Etevaldo dos Santos Costa, Francisco Barbosa-Junior, Filipe Andrade Bernardi, Tiago Lara Michelin Sanches, Lariza Laura de Oliveira, Vinicius Costa Lima, Newton Shydeo Brandão Miyoshi, Domingos Alves

**Affiliations:** 1 Universidade de São Paulo, Instituto de Ciências Matemáticas e de Computação, São Carlos, SP, Brasil.; 2 Universidade de São Paulo, Instituto de Física de São Carlos, São Carlos, SP, Brasil.; 3 Universidade de São Paulo, Faculdade de Medicina de Ribeirão Preto, Departamento de Medicina Social, Ribeirão Preto, SP, Brasil.; 4 Universidade de São Paulo, Programa de Pós-Graduação em Bioengenharia, São Carlos, SP, Brasil.

**Keywords:** COVID-19, Sars-Cov-2, Pandemic, Brazil

## Abstract

**INTRODUCTION:**

Five months after the first confirmed case of COVID-19 in Brazil, the country has the second highest number of cases in the world. Without any scientifically proven drug or vaccine available combined with COVID-19’s high transmissivity, slowing down the spread of the infection is a challenge. In an attempt to save the economy, the Brazilian government is slowly beginning to allow non-essential services to reopen for in-person customers.

**METHODS:**

In this study, we analyze, based on data analysis and statistics, how other countries evolve and under which conditions they decided to resume normal activity. In addition, due to the heterogeneity of Brazil, we explore Brazilian data of COVID-19 from the State Health Secretaries to evaluate the situation of the pandemic within the states.

**RESULTS:**

Results show that while other countries have flattened their curves and present low numbers of active cases, Brazil continues to see an increase in COVID-19 patients. Furthermore, a number of important states are easing restrictions despite a high percentage of confirmed cases.

**CONCLUSIONS:**

All analyses show that Brazil is not ready for reopening, and the premature easing of restrictions may increase the number of COVID-19-related deaths and cause the collapse of the public health system.

## INTRODUCTION

With 13 million cases and 576,000 deaths, the largest pandemic since the Spanish Flu in 1918 has now reached over 200 countries in the world. COVID-19, a disease caused by Sars-Cov-2, started in Wuhan but rapidly spread across Asia, Europe, and America^1^. Brazil, which had its first confirmed case on February 25, 2020, almost two months after the announcement of the outbreak of the disease in China, is now the country with the second highest total number of confirmed cases^2^ ^,^ ^3^. By July 14, 2020, we accounted for 1,888,000 cases and 72,000 deaths^1^.

As a new disease, despite all advances in technology and rapid genome analysis, there are no scientifically proven treatments, such as drugs and vaccines, to protect people or cure infected patients. Thus, the main recommendations from the World Health Organization (WHO) for the general population are to maintain social distance and hygiene habits such as the use of alcohol-based hand sanitizers and face masks, measures intended to slow the spread of the virus^4^.

Furthermore, social inequalities in Brazil highlight the importance of higher isolation rates since the public health system cannot withstand high pressure. SUS, the Brazilian Public Health System, is responsible for the support of 80% of the population and could collapse if a considerable number of people contract the disease in a short period of time^5^.

Moreover, people’s confidence in the government is referenced as one of the critical points in handling the pandemic successfully. Nevertheless, Brazil is going through a hard time in the political sphere. As examples that directly impacts the COVID-19 battle, the health minister was replaced twice in two months and the country’s president has been frequently seen in groups not complying with the medical community’s recommendations, thereby influencing the population^6^ ^,^ ^7^.

Understanding data is essential for the proper care of the population. Therefore, in this study, we analyze the situation of the pandemic in other countries to explore how they dealt with the disease and under which conditions they decided to resume non-essential services^8^. By understanding the evolution of each country and the statistics in Brazil, the goal of this study is to evaluate whether the tropical country is gathering knowledge from other regions and easing restrictions according to certain metrics such as the decrease in daily cases and deaths.

## METHODS

This article is a descriptive observational study using classical statistical metrics, such as frequency and percentage. The analysis is divided into two parts. First, the aim was to characterize how Brazil is evolving in the COVID-19 war. For this, the Brazilian data at the federal level were compared with data from other countries regarding three types of information: the number of new cases, the number of new deaths, and active cases. For this analysis, we collected data from John Hopkins^9^, which allowed us to obtain the daily and total cases, deaths, and recovered patients, along with the evolution of the pandemic. Active cases are not directly obtainable from the Hospital database; thus, we computed them using the following equation: *active_cases(x) = total_cases(x) - total_deaths(x) - total_recoveries(x)*, where *total_cases(x)*, *total_deaths(x)*, and *total_recoveries(x)* are the total number of positive cases, deaths*,* and recovered patients, respectively, until day *x.* The measure is a good track to understand how many patients are currently transmitting the viruses or might require hospitalization, which is important for public health policies. 

Furthermore, we also compared the total number of cases in each country according to their size. The relative comparison is essential because countries with different sizes probably have more cases compared to smaller regions. Therefore, by dividing the cases by the population size, one can understand the proportion of inhabitants of each country that contracted COVID-19 and the differences and similarities among couples of countries. Besides Brazil, seven countries are evaluated: South Korea, the United States of America, Spain, Italy, New Zealand, Germany, and China.

Along with the comparison of countries, we also compared the first and second positions in the worldwide rank of case numbers provided by^1^. To do this, we used the epidemiological knowledge that virus propagation within the population can fit an exponential model^10^. Thus, we used computational simulations in Python to fit the total number of cases of Brazil and the US as an exponential curve. To perform this analysis, we used the data from the previous two weeks, the approximate period of time Sars-Cov-2 remains in an individual organism^11^, to obtain the exponential parameters. Then, we projected the data for the next 15 days. 

Another statistic is the testing rate for each confirmed case. Testing suspected cases reduces the under-detection probability and enhances the reliability of the data. Data were retrieved from Our World in Data^12^ for each country. The results are shown in Section 3.1. 

Finally, in Section 3.2, we seek to understand how COVID-19 performed within the Brazilian territory. Disaggregating geographically by state, the analyses aimed to characterize the number of cases, the number of deaths, and their influences on population rates over the time that the virus disseminated throughout the country over 5 months (from February to July). The behavior of dissemination and danger of the virus was characterized by the contamination rate, which describes the speed of spread of the virus through the number of cases normalized by the population of each place per 100 thousand inhabitants, and the lethality rate, which describes the proportion of people who die among all those infected^13^.

 Data were obtained from the State Health Secretaries’ databases and along with information of absolute and relative cases for each of the main states in Brazil, we also evaluated the spatial evolution of the pandemic in the state of São Paulo, the most populated location in Brazil. Finally, we discuss the evolution of reopening measures in Brazil, showing a map with coping policies for each country. The coping information was retrieved from the news and collected from https://ciis.fmrp.usp.br/covid19/.

## RESULTS

### Brazil and the world

As previously mentioned, it took two months for Brazil to have its first confirmed case of COVID-19, which gave the country a foundation of information and knowledge to better understand the evolution of the disease, as well as the opportunity to see how other countries proceeded with reopening.

**FIGURE 1:**
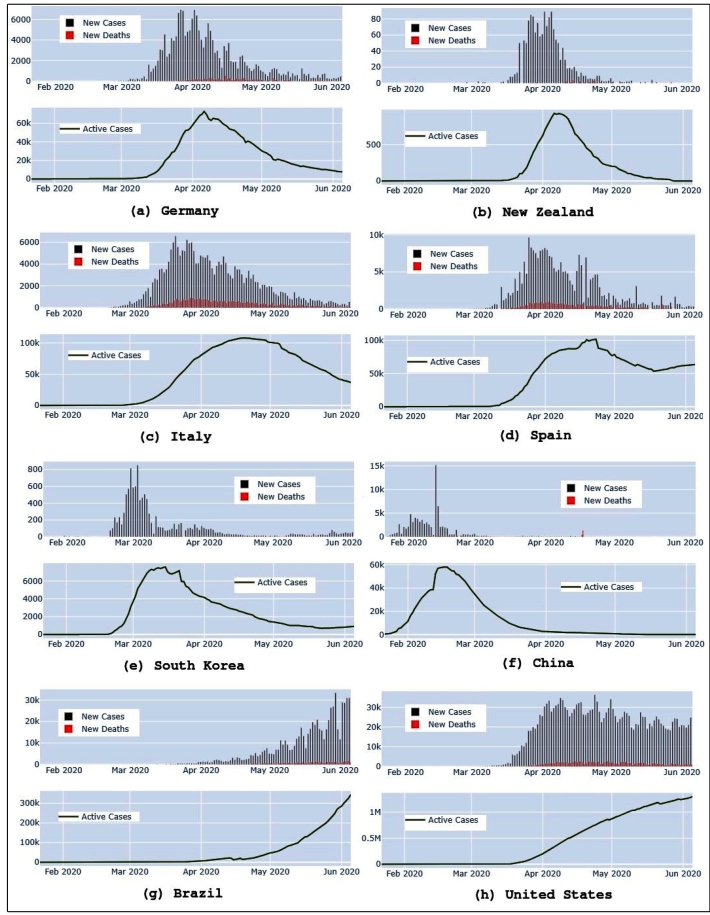
Eight important countries in the fight against COVID-19. The graph shows the number of daily cases and new deaths as well as active cases for each location. Although all countries have been easing their restrictions, Brazil and the United States notably do not follow the same patterns as other regions and present a high number of active cases.

First, one can start by understanding the situation in Wuhan, the breakpoint of the pandemic that previously suffered from SARS and the Avian flu^14^. Despite the recent history of respiratory diseases, COVID-19 is a new challenge that is not yet fully described by science, and the prognosis of patients can be unpredictable. In order to contain the spread of the disease and a high number of deaths, the Chinese government ordered a lockdown in Hubei, applying severe restrictions regarding social isolation which were widely obeyed^1^. Furthermore, the culture of using face masks as a routine probably helped to decrease the rate of transmission^15^. By the beginning of March, China had already less than 100 new cases per day, and, by mid-June, there were under 10 new cases per day^1^. The country was one of the first to reopen and is currently tracking new cases by applying social distancing in workplaces, constant disinfection, and temperature measuring more than once a day^16^ ^,^ ^17^. These actions appear to be effective, considering the number of new cases shown in Figure 1f.

New Zealand is another country that has succeeded in beating COVID-19. After 24 days with no detection of COVID-19, a patient was diagnosed on June 16, having contracted it in the United Kingdom, meaning no local transmission had occurred in three weeks^18^. The reason for such success? Travel restrictions and quarantine measures with a low level of confirmed cases^19^. Prime Minister Jacinda Ardern has demonstrated strong leadership, convincing almost five million people to respect the stay-at-home guidelines^20^. Furthermore, testing and tracking were applied all over the country with the support of mobile applications that use QR codes to scan buildings and alert possible infected people^21^. After one week with zero new cases, New Zealand began to ease its restrictions^22^.

South Korea also used technology, isolation restrictions, and extensive temperature measuring^23^ ^,^ ^24^. In Europe, Germany also applied consistent rules and strategies to fight the virus, such as intensive care unit (ICU) monitoring, testing, and patient tracking^25^ ^,^ ^26^. Italy was one of the countries most affected by COVID-19 due to a delay in establishing restrictions in many points of the country. The ‘Milan does not stop’ campaign, for example, ignored the isolation instructions from WHO in an attempt to prevent the economy from collapsing^22^. After losing control of the disease spread, a lockdown began on March 9 and lasted until May 18^27^ ^,^ ^28^.

Although these countries may have established different strategies, most of them relied on WHO instructions, scientific evidence, and strong leadership. Figure 1 shows that almost all countries are on the downside of the curve of new cases. The database of the graphs was retrieved from the John Hopkins Hospital^9^. The number of active cases in Italy, around 13,000, is still high, which requires intense observation of health services capacity since isolation restrictions are being loosened. With no drug or vaccine, all countries must keep monitoring its cases to understand whether it is time to re-apply restrictions or if one proceed to new phases of reopening.

However, one can see that Brazil and the United States, the second and first countries, respectively, in the number of confirmed cases, do not follow the same patterns in the new cases/deaths and active case graphs, as shown in Figures 1g and 1h. Furthermore, the graph of active cases shows a steep incline, while new cases and deaths are increasing.

**FIGURE 2:**
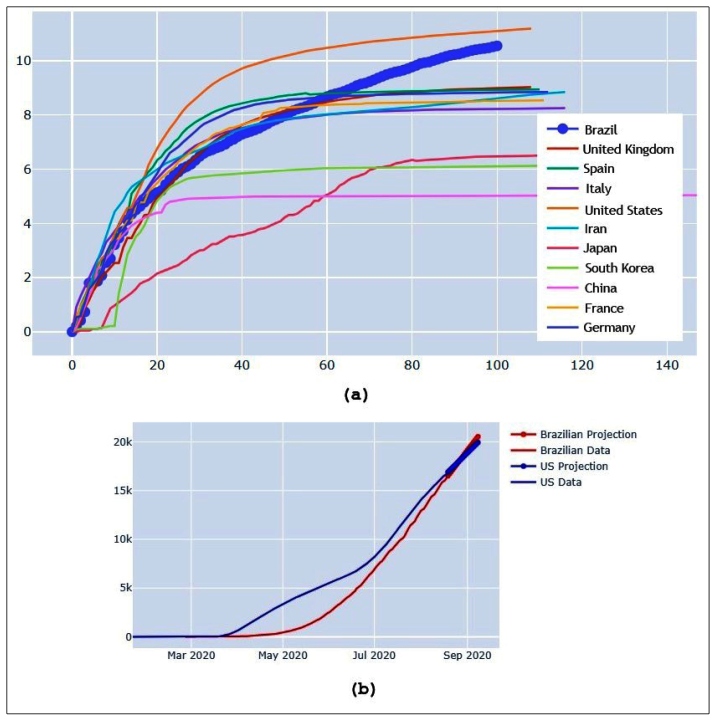
(a) Brazil ranks second in total number of cases worldwide and continues to grow. However, (b) by the end of August, Brazil is expected to overtake the US in the total number of cases relative to population size.

This is also evident in Figure 2a, where most countries’ curves are flattening and Brazil and the United States curves continue to increase. As the US and Brazil rank as first and second in numbers of active cases^1^, we compared both exponential projections in an attempt to predict the future. In Figure 2b, it appears that Brazil will overtake the US in numbers of active cases by the end of August. Such analysis considers the relative number of cases in both countries to remove population size influence. This means that besides being the country in South America with the most COVID-19 cases, Brazil will also have the highest number of cases worldwide. 

**FIGURE 3:**
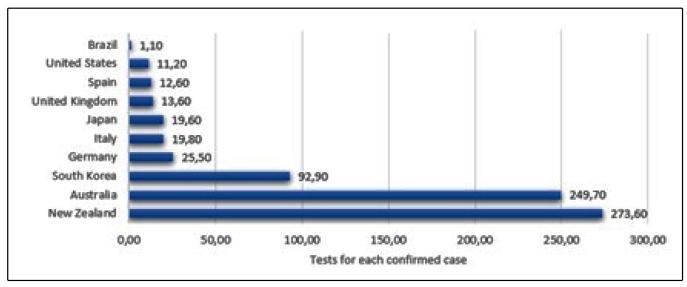
Besides the high number of cases in Brazil, the country is known for its lower testing rate. This has direct consequences on the under detection COVID-19, meaning that Brazil possibly has more cases than those noted by health care centers. Conversely, South Korea and New Zealand are among the countries with the highest rates of testing and are considered successful cases in the pandemic.

The situation is even more worrying, as Brazil has a low test rate and, consequently, a high likelihood of under detection (Figure 3) compared with countries that are coming out of lockdown. Furthermore, several places in Brazil are easing isolation rules and opening non-essential services, such as malls, which are usually environments with low air circulation and, therefore, conducive to the spread of the virus.

It should be taken into account that Brazil is a very heterogeneous country with high social inequality. The pandemic is affecting the public health system and the population in a non-uniform way, as approximately 11.4 million people live in shanty houses. The following section highlights these differences and shows more in-depth features of this enormous country.

### Inside Brazil

**FIGURE 4:**
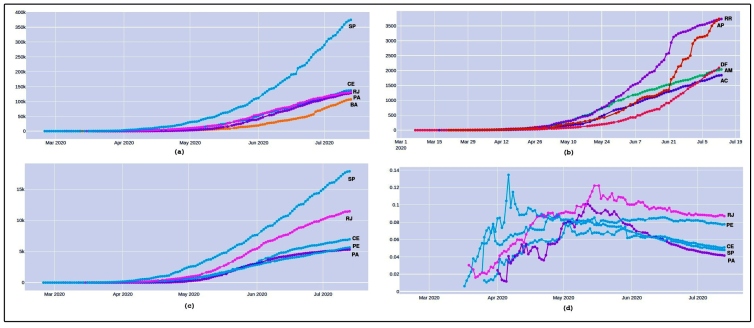
(a) Evolution of COVID-19 cases in the five Brazilian states with the highest number of cases. The first position is São Paulo (SP), a highly industrialized area with 45 million people, followed by Ceará (CE), Rio de Janeiro (RJ), Pará (PA), and Bahia (BA); (b) five top states with high contamination rates: Amapá (AP), Amazonas (AM), Acre (AC), Roraima (RR), and Ceará (CE). Most states are located in the North region of Brazil, a location with a low percentage of ICU beds and high need for medical resources such as mechanical ventilators; (c) evolution of COVID-19 deaths in the five Brazilian states with the highest number of deaths: São Paulo (SP), Rio de Janeiro (RJ), Ceará (CE), Pernambuco (PE), and Pará (PA). Pernambuco appears for the first time in the analysis and shows, along with Ceará and Pará, the fragility of the North and Northeast regions of Brazil; (d) the five Brazilian states with the highest lethality rate of COVID-19: Rio de Janeiro (RJ), Pernambuco (PE), Ceará (CE), São Paulo (SP), and Pará (PA).

Brazil, with 26 states and one federal district, is the seventh most populated country, and the fifth with the greatest territorial extension worldwide^29^. Therefore, the country is inconsistently dealing with the coronavirus epidemic. Each Brazilian state behaves like a small country, and the segregation of analysis aims to strengthen the action plans. The following analyses considered the five Brazilian states with the highest numbers in each indicator. Figure 4a shows the accumulated number of cases over time. In order, the five states with the highest number of cases were São Paulo, Ceará, Rio de Janeiro, Pará, and Bahia. At the end of May, they recorded approximately 880,000 cases, 47% of all Brazilian cases. São Paulo has been the leader in the number of cases since the beginning, with approximately 173% more cases than the second ranked state, Ceará. This is because São Paulo is the industrial center and the most populated area of the country, with approximately 45 million people.

However, the absolute number of cases alone does not provide a complete picture of the severity and speed of dissemination. Thus, Figure 4b shows the contamination rate. The latter four states did not have a large accumulated number of cases, but showed high daily growth in relation to the other Brazilian states.

A different scenario is observed when analyzing the contamination rate, as shown in Figure 4b. The measure refers to the number of cases normalized by the population of each state per 100 thousand inhabitants. Since the end of April, the states of Amapá, Roraima, Amazonas, and Acre have presented high community transmission of the coronavirus. This has resulted in a new focus on the North region of Brazil. None of these regions were even mentioned in previous analyses. This negative impact of this statistic was clearly seen in mid-April when the state of Amazonas became the first Brazilian state to be on red alert, asking for international support due to the collapse of the funeral and health system^30^ ^,^ ^31^. With the diversity and immensity of the country, Brazil has several areas and different needs that require attention. Thus, it is essential to explore different indicators to capture these needs as quickly as possible.

Figure 4c presents the death analysis. It is expected that the number of deaths will be proportional to the number of cases. Four of the states with the highest absolute number of deaths have already been mentioned as critical in previous analyses; however, the state of Pernambuco is unprecedented and had more than 2,500 deaths by the end of May. Together, the five states with the highest number of cases have reported almost 20,000 deaths and represent 75% of total Brazilian deaths.

Regarding daily case information, the highest number in the country occurred in São Paulo on June 3 when 609 deaths due to COVID-19 were recorded in 24 hours. Hospital morgues located in Rio de Janeiro, which also has a high number of daily deaths, are dealing with overcrowding and accumulating bodies day after day^32^.

The last indicator explored in this subsection is the lethality rate, as shown in Figure 4d. The lethality rate is the ratio between the number of deaths and the total number of cases of a disease in a given period; it represents the risk that people with the disease have of dying from the disease^13^. 

## DISCUSSION

Brazil has a high rate of COVID-19 transmission. Within 15 days, its number of cases had doubled, which translated to 500,000 new cases^9^. The heterogeneity and extensiveness show a burdensome scenario within the decision-making process and the need for protective measures. Social inequalities enhance the necessity of deep analysis prior to easing restrictions based on intensive care units, infrastructure, and infection rates presented in Section 3. For instance, the North region required significant medical support and structure in order to prevent a worse collapse in the health system. The Northeast also showed high lethality and death rates, mainly in Ceará and Pernambuco. São Paulo and Rio de Janeiro appear in almost all COVID-19 analysis indicator rankings. Both states represent the main economic poles in the country, and the high density of people in both areas highlights the importance of continuous tracking and testing. 

The Brazilian government does not seem to be learning lessons from other countries, such as those referenced in Section 3.1. As previously mentioned, all of them, except for the United States, only eased restrictions after intensive tracking, testing, and a descending curve in the number of daily cases was shown. São Paulo and Rio de Janeiro, along with other states in Brazil, do not follow the same approach (see Figure 3a). In addition, the number of active cases is still high, along with all other indicator analyses concerning each state. 

This is an urgent issue and must be addressed in all public spheres so that we can improve our response to the pandemic. In public health, prevention is always the least costly option, especially in this case, where an individual’s response to the infection could be death. Supported by the quantitative information in Figure 2b demonstrating that Brazil is expected to overtake the US in total number of cases (proportionally by population size), the current low isolation rates, low population testing percentage with consequently high under detection, high daily cases, and death rates all suggest that Brazil has eased its restrictions prematurely and may face severe consequences in the near future as a result, such as becoming the next global epicenter of the pandemic.
